# Refractive Index Sensor Based on Double Side-Polished U-Shaped Plastic Optical Fiber

**DOI:** 10.3390/s20185253

**Published:** 2020-09-14

**Authors:** Shumin Wang, Daming Zhang, Yan Xu, Siwen Sun, Xiaoqiang Sun

**Affiliations:** State Key Laboratory of Integrated Optoelectronics, College of Electronic Science & Engineering, Jilin University, Changchun 130012, China; wangsm18@mails.jlu.edu.cn (S.W.); zhangdm@jlu.edu.cn (D.Z.); xyan17@mails.jlu.edu.cn (Y.X.); sunsw18@mails.jlu.edu.cn (S.S.)

**Keywords:** plastic optical fiber, U-shaped, side polish, sensor

## Abstract

A U-shaped double-side polished plastic optical fiber (POF) is demonstrated as a liquid refractive index (RI) sensor. The refractive index of glycerinum solutions is identified by the intensity detection on the bending and evanescent wave loss change. Heat treatment and mechanical polishing are adopted to form the symmetrical side-polished POF probe. The processing parameters are experimentally optimized on the power transmittance. The sensitivity of 1541%/RIU (Refractive Index Unit) can be obtained with a resolution of 5.35 × 10^−4^ in the scope of 1.33–1.39. The favorable temperature characteristic is proved to offer stable RI sensing from 20 to 50 °C. This simple POF sensor has potentials in low-cost visible light intensity RI detection.

## 1. Introduction

As a basic optical parameter, the refractive index (RI) can be widely used to identify chemical substances in many analytical applications, including food safety [1], medical examination [2,3], and environmental monitoring [4,5]. In the past few decades, the fiber-based RI sensor has been extensively explored because of its advantages of low cost, multiplexing capability, and fast response. Thus far, various fiber optic sensors have been proposed, including multi-mode interference [6], fiber Bragg grating [7], long period fiber grating [8], and so on. However, in practical applications, these sensors usually require expensive equipment, complex manufacturing processes, and high production costs. In recent years, plastic optical fiber (POF) has received more attention because of its superior performance in machinability, robustness, optical coupling and handling compared with silica fiber [9,10,11,12,13]. Generally, structural modification methods such as tapering [14,15,16], side polishing [17,18,19], and making a hole [20,21] are used to modify POFs to improve RI sensitivity. Due to the multi-model characteristics, POF based RI sensor is more suitable for intensity modulation schemes [22,23,24]. Multiple solutions should be combined to improve the sensitivity, such as the shape, working wavelength, and surface modification. Operating in visible light, the change of light intensity caused by different refractive index can be intuitively recognized and detected [25,26]. Among these solutions, the U-shaped POF sensor allows the light source and collector to be placed on one side, which offers the feasibility of dip-type probe. Bending and side polishing the fiber may reduce the number of modes and convert the lower order modes into higher order ones, increasing the power of the evanescent wave [27].

In this paper, a U-shaped double side-polished POF is proposed and experimentally demonstrated. The mode propagation and loss characteristic of a sensor probe have been investigated. The bending radius, polished depth, and deviation angle are optimized to provide better sensitivity. The heat treatment and mechanical processing is adopted in the fabrication of the U-shaped POFs probe. The symmetrical double side-polishing allows excess contacts of POF cores with the liquid analyte, which is favorable to better resolution. Tests at different temperatures prove the favorable thermal stability. Visible light operation promised potentials of this POF sensor in high absorption liquid RI sensing.

## 2. Principle

**Figure 1:**
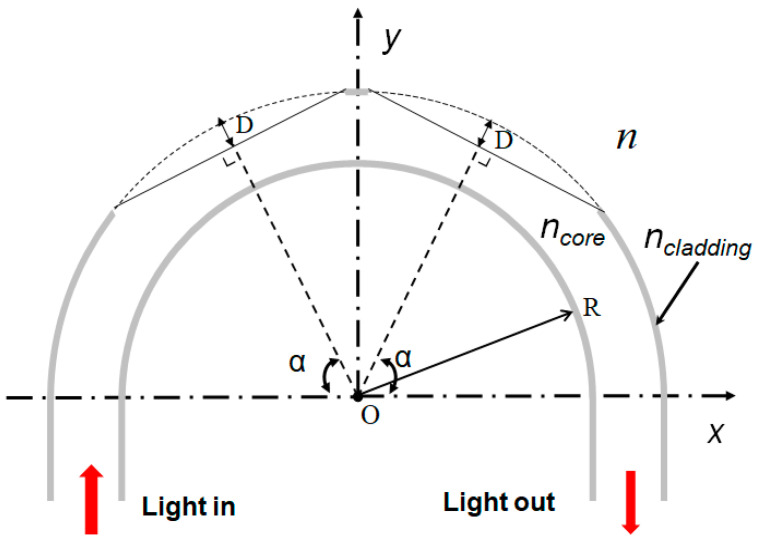
Schematic of a plastic optical fiber (POF) probe with a U-shaped and side-polished structure. n—refractive index (RI) of the environmental media. R—the curvature radius. D—the polished depth, α—the polished position (angle).

The U-shaped and dual side-polished structure probe is shown in Figure 1 below. The POF is bent to a U-shape with a curvature radius R. The polishing position is determined by the angle α. The polished depth D affects the contacting area of evanescent wave (EW) to the sample medium. Here, *n* represents the RI of environmental media, and *n*_core_ is the RI of POF core. For the multimode POF, different modes that propagate along the fiber have different transmission characteristics, the modes propagating in a fiber play an important role for favorable sensing performance [28]. The sensing performance of proposed probe is mainly determined by the refraction loss and EW absorption. Though the incident angle of a ray is maintained as propagating along a straight optical fiber, it will be changed to a U-shaped and side-polished POF. Rays traveling at various angles or different optical modes will be affected diversely. Only the rays that satisfy the critical angle conditions are supposed to continue propagating to another end of the bend section, which results in the loss of the rays initially guided by the fiber.

According to Ref. [27], the RI of the outer curvature of a U-shaped fiber with low bend radius will be significantly lower than that of the core. Then, the rays propagating at the outer curvature refract at the core/analyte interface, resulting in high refraction loss. Moreover, when the outer curvature of the U-shaped POF is removed by polishing, the RI difference between the analyte and the inner curvature of fiber will decrease, leading to the increment of evanescent field power, as well as its penetration depth. These structural modifications are used to provide better sensing performance.

Here, the sensitivity S is firstly defined by the slope of optical power transmission within the detection range [23]:

S(%/RIU) = ∆T/∆n(1)

where the transmittance T = Pa/Pd, Pa, and Pd are the average light output power of the POF sensor when the U-shaped probe is immersed in the analyte solution and deionized water, respectively. ΔT is the change of the output optical power and ∆n corresponds to the RI change of liquid analyte. The resolution (R) of the proposed sensor is further defined as [24]:

R = 3σ_a_/S (2)

where σ_a_ is the standard deviation of transmittance when the POF sensor probe is exposed to the blank analyte (air). It should be noted that the POF sensor probes with different structural parameters have different σ_a_. In this work, σ_a_ is calculated by:

(3) $$\sigma_{a}=\sqrt{\frac{\sum_{i=1}^{n}{\left(x_{i}-\bar{x}\right)}^{2}}{n-1}}$$

where *x_i_* is the detected optical power output from the sensor probe when it is exposed to the air and $\bar{x}$ is the average value of *x_i_*. For better accuracy, *n* is chosen to be 10. Before each test, the POF sensor probe is firstly immersed into the deionized water with a RI of 1.33, then the probe is thoroughly cleaned and dried.

## 3. Fabrication and Characterization

**Figure 2:**
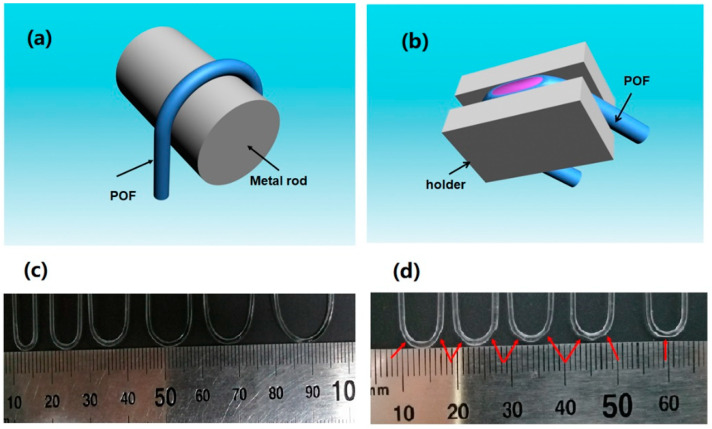
Fabrication process and pictures of U-shaped double side-polished POF sensors. (**a**) schematic of U-shape formation by thermal setting method (heating the metal rod), (**b**) fabrication of the U-shaped double side-polished POF probe with varied polishing depth and angle, (**c**) U-shaped structure with a radius of 2, 3, 4, 5, 6, and 7 mm, (**d**) U-shaped structure with a radius of 3 mm, polished depth of 300 µm, and side polished angles of 30, 45, 60°, 70°, and 90°. The red arrow in the picture indicates the polished surface.

**Figure 3:**
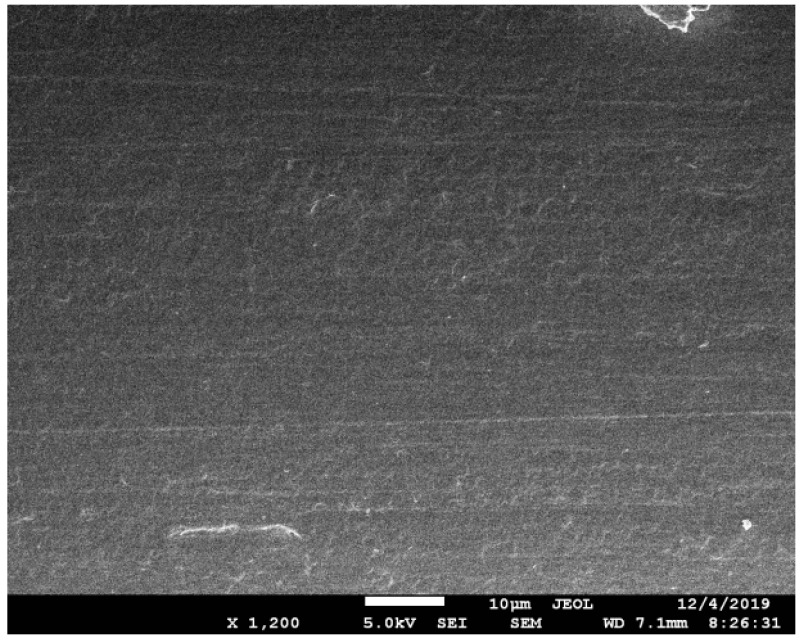
Surface status of side-polished POF after the fine polishing.

The POF used here is the commercially available step-index POF (Chongqing Shijizhiguang POF Co., Ltd., Chongqing, China). The Polymethyl methacrylate (PMMA) core of POF is with a diameter of 980 µm and an RI of 1.49. The thickness and RI of the fluoro resin cladding are 10 µm and 1.41, respectively. As shown in Figure 2a,b, the POF primarily rotates around a steel cylinder, and is then shaped by heat setting. A U-shape probe with a small bend radius is obtained. Next, grinding papers are used to symmetrically polish the bent POF, during which the POF is supposed to be fixed well to guarantee polishing precision. The side-polishing includes rough polishing and fine grinding. A rough polishing paper with larger particles was primarily adopted to remove the side part of POF core to approximately form the desired profile of probe. Next, a fine emery paper with smaller particles was used to further reduced the roughness of exposed area. The quality of the polished surface was then checked by scanning electron microscope (SEM) characterization. As shown in Figure 3 below, no obvious particle or roughness is observed. Considering the visible light operation, this polishing process is convinced to provide quantified surface for sensing test.

The depth and the angle (or position) of side polishing can be tuned through altering the exposed surface on the holder. The fabricated U-shape POF probes with different radii of 2, 3, 4, 5, 6, and 7 mm is shown in Figure 2c. The polished U-shape structures with a radius of 3 mm, polished depth of 300 µm, and side angles of 30°, 45°, 60°, 70°, and 90° are shown in Figure 2d.

**Figure 4:**
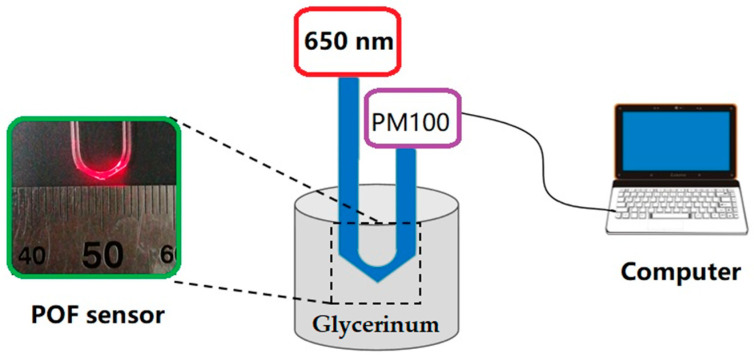
Schematic diagram of POF sensor measurement setup. Light from 650 nm laser source is coupled into the POF, and the output light is detected by the optical power meter PM 100.

The measurement setup to record the transmittance of POF probe when it is immersed in liquid analyte is illustrated in Figure 4. Glycerinum solutions with different RI ranging from 1.33 to 1.42 were prepared before test. The RI step of the neighboring glycerinum solutions is 0.01. The 650 nm red light from a laser source (MRL-III-655L-100 mW) was coupled into the POF probe. An optical power meter (PM100D) was adopted to record the transmitted power. Tests were conducted at 20 °C to avoid the impact of temperature on the RI test accuracy.

Though the collimated light produced by a laser does not necessarily fill all possible modes a POF can guide, lower order modes can evolve into higher order modes in the U-shape POF probe [12]. Considering that the working principle of proposed sensor is based on the U-shape bending loss and evanescent field, lasers with better stability and strong anti-interference ability can compensate the deficiency of intensity detection that the measurement results are easily affected by the fluctuation of a light source. The errors and limitation on the resolution can be effectively restrained. If white LED or red LED was applied in the test, more higher order modes are expected to be excited in the U-shape POF probe and more power leaks at the polishing position. Better sensing performance may be expected according to the definition of sensitivity and resolution. However, the optical spectrometer is then supposed to be used to obtain the RI change information due to the wider spectrum of LEDs. To be noted, the signal noise ratio may deteriorate due to the increased refraction loss, which will reduce the detection range.

## 4. Results and Discussion

### 4.1. Effect of Curvature Radius

**Figure 5:**
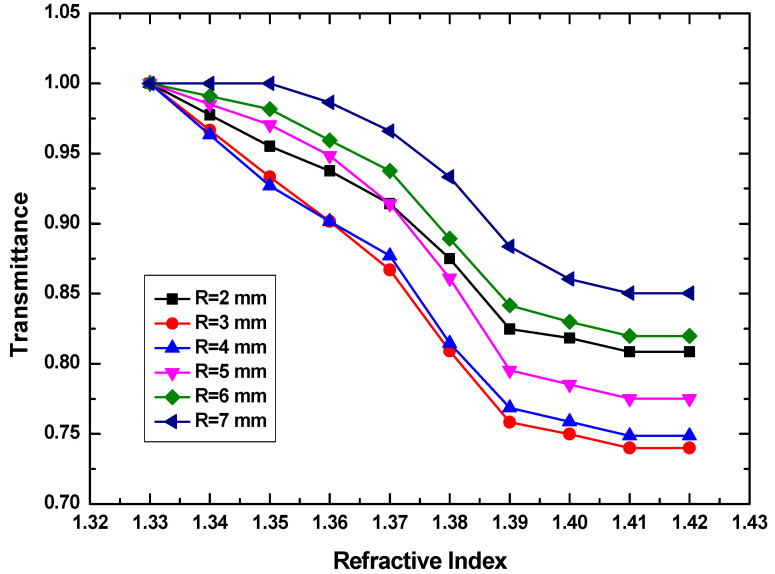
Transmittance as a function of RI of glycerinum solution at bending radius from 2 to 7 mm.

The normalized measured transmission of the fabricated RI sensor as a function of bending radius (R = 2, 3, 4, 5, 6, 7 mm) is shown in Figure 5. Obviously, the sensitivity increases as the bending radius is decreasing. This can be explained that the interaction between the fiber mode and the external environment is enhanced when more light leaks from POF. However, a smaller bending radius does not always lead to a better sensing performance. The sensitivity drops as the bending radius is smaller than 3 mm, because the refractive loss of probe with a diameter of 2 mm is so large that the output signal is too small to provide a favorable signal-to-noise ratio. There exists a lower limit for the bending radius. The best sensitivity of 417%/RIU is observed at R = 3 mm. As mentioned above, the core and cladding RI of POF used in this work are 1.49 and 1.41, respectively. When the RI of glycerinum solution is over 1.41, the propagation loss keeps no change, leading to a cut-off of sensitivity. Therefore, the sensitivity is obtained from the linear RI change region from 1.33 to 1.39. When the RI of glycerinum solution is larger than 1.39, the normalized transmission of U-shaped POF sensor is as sensitive as it is in lower RI range. This can be attributed to the power saturation, because the RI increasing of glycerinum solution implies the reduction of beam reflection at the analyte/PMMA core interface, this effect occurs as the outside RI approaches the core RI. Therefore, in the following investigation, R is selected to be 3 mm.

### 4.2. Effect of Polishing Position

**Figure 6:**
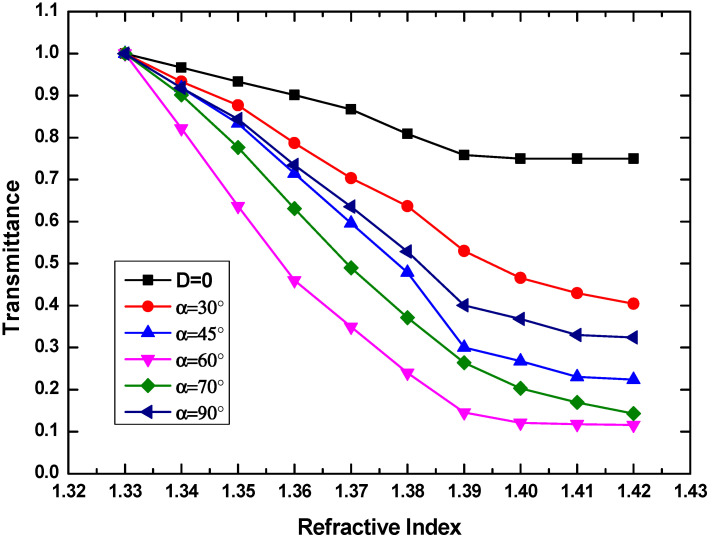
Transmittance as a function of RI glycerinum solution when the side throw angle ranges from 30° to 90°. Here, the bending radius and polishing depth are fixed at 3 mm and 300 µm, respectively.

The POF with a bending radius of 3 mm is side-polished symmetrically at different polishing angles of 30°, 45°, 60°, 70°, and 90°. Here, the polishing depth is fixed at 300 µm. α = 90° refers to only one top polishing. The transmission power of POF sensor as a function of side-polished angle is shown in Figure 6.

Compared to the sensing performance without side polishing, more remarkable output change can be observed when the polishing angle is increasing, which implies a higher sensitivity. The maximum sensitivity of 1417%/RIU happens at 60°. The sensitivity decreases when 0° < α < 60° or 90° > α > 60°. Of note, the linear response region shrinks with the increment of polishing angle when α < 60°, while the linear response region enlarges with the increment of polishing angle when α > 60°. The sensitivity and resolution from Figure 6 are shown in Table 1. It can be seen that the minimum σ_a_ of 2.72 × 10^−3^ can be obtained at α = 30°. However, the best resolution of 7.89 × 10^−4^ appears at the maximum sensitivity of 1417%/RIU, when α is 60°. Therefore, α is chosen to be 60° in this design. However, above results just comes from the fixed polishing depth of 300 µm, which deserves to be further optimized to get a better sensing performance.

### 4.3. Effect of the Polished Depth

**Figure 7:**
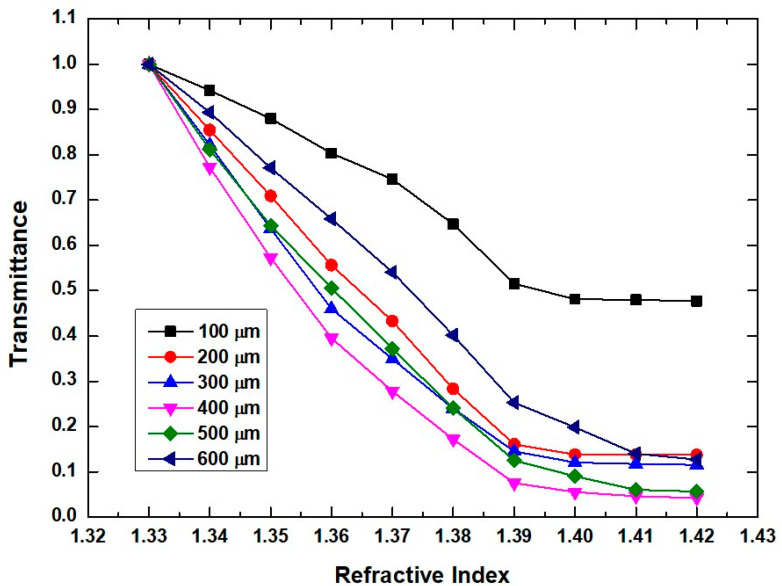
Transmittance as a function of polishing depth when the side throw angle and bending radius are 60° and 3 mm, respectively.

The polishing depth is further investigated when R = 3 mm and α = 60°. The transmittance as a function of RI at different polishing depths of 100, 200, 300, 400, 500, and 600 µm is shown in Figure 7. It can be seen that the sensitivity monotonously increases, when the polishing depth is lower than 400 µm. Further increasing the polishing depth, the sensitivity will decline obviously. This may be explained that the shrinking of fiber diameter caused by the deeper polishing changes the critical angle of reflection at the analyte/core interface. Then, more light will be reflected back to the fiber and propagate along the fiber, without leaking out the outer space. The transmittance increasing weakens the power modulation effect induced by the glycerinum solutions, which leads to the reduction of sensitivity. The detailed test results are listed in Table 2. When a favorable standard deviation of transmittance σ_a_ = 2.75 × 10^−3^ is obtained, the maximum sensitivity of 1541%/RIU and the best resolution of 5.35 × 10^−4^ can be observed at D = 400 µm. Considering the improvement of sensitivity as a key characteristic of sensor, D is chosen to be 400 µm.

To be noted, in this case of U-shaped double side-polished multimode POF, it is hard to formulate a precise analytical framework due to the abrupt changes in the direction of light propagation in the highly deformed bend region, and the inhomogeneity in both material and the geometrically defined numerical aperture. Thus, the ray tracing method has been used to study the light propagation characteristics. The simulation results exhibit similar random behaviors to those in the measured transmittance at different bending radii, polishing angles and depths, which may be attributed to the complex physical effect of refractive loss and evanescent wave absorption.

### 4.4. Effect of the Temperature

**Figure 8:**
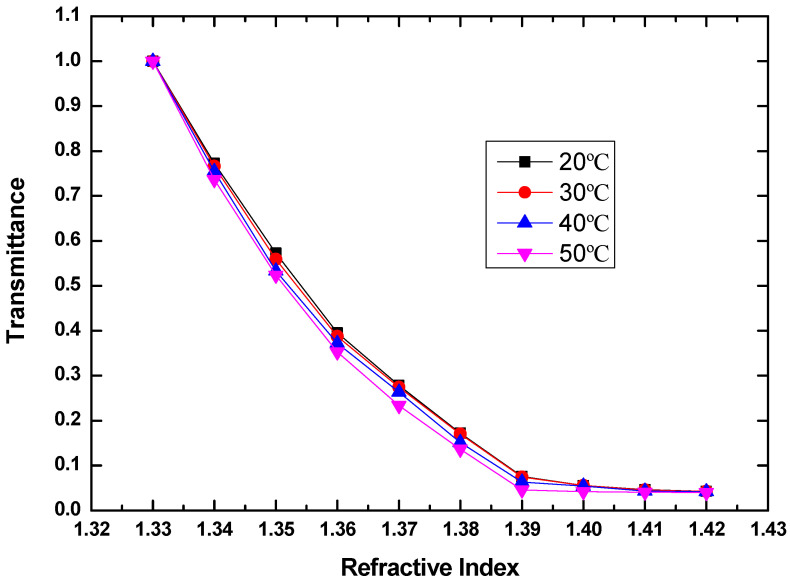
Measured transmittance as a function of RI of glycerin solution at temperatures of 20, 30, 40, and 50 °C, when the bending radius is 3 mm, the polished angle is 60 degrees, and the polished depth is 400 um.

Normally, the RI sensor is supposed to operate at a constant temperature. However, the RI variations of the POF probe, as well as the structure parameters would have influence on the sensor performance when the ambient temperature changes. Therefore, the temperature influence to the POF RI sensor deserves to be investigated [29,30,31]. In this work, the analytes are glycerinum solutions with different concentrations prepared at 20 °C. By varying the concentration of the glycerin solution, RIs ranging from 1.33 to 1.43 with step of 0.01 can be obtained. These solutions have been characterized by a commercial Abbe refractometer and described by:

n = 1.333 + 0.13 C (4)

where C is the volume concentration of glycerol. The temperature of glycerin solution has been changed from 20 to 50 °C at a step of 10 °C by a digital temperature controller connected to a heater. A thermometer was immersed into the liquid to monitor the practical temperature. The transmittance as a function of RI at different temperatures of 20, 30, 40, and 50 °C is shown in Figure 8.

**Figure 9:**
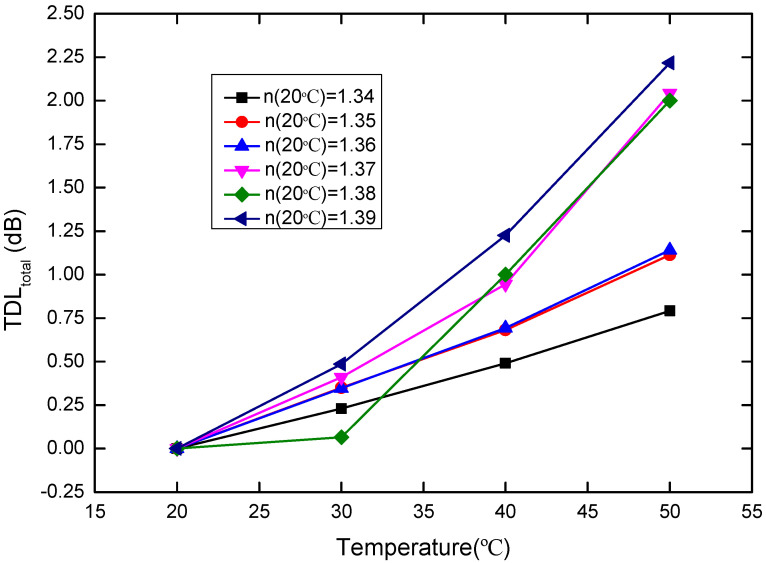
TDL_total_ versus temperature (20–50 °C) for the sensor probe, where the RIs of the glycerin solutions with different concentration at 20 °C are 1.34, 1.35, 1.36, 1.37, 1.38, and 1.39, respectively.

To investigate the temperature influence, the propagation loss of the sensor induced by the temperature variation from 20 °C refers the total temperature dependent loss (TDL_total_):

TDL_total_ = Loss_T°C_ − Loss_20°C_ (5)

where T is the temperature. In accordance with Figure 8, TDL_total_ as a function of temperature is shown in Figure 9. TDL_total_ will lead to the RI deviation. Here, this deviation is referred to as the total temperature-dependent RI (TDR_total_), which includes two parts. One is the TDR induced by the thermo-optic effect and thermal expansion effect of the sensor probe (TDR_sensor_). Another TDR is induced by the thermo-optic effect of the measured liquid (TDR_liquid_). Therefore, the TDR_total_ can be expressed as:

TDR_total_ = TDR_sensor_ + TDR_liquid_ (6)

**Figure 10:**
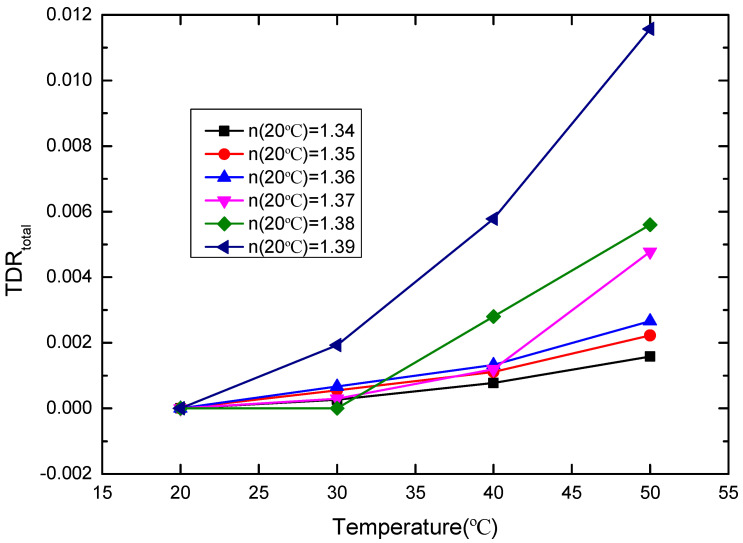
TDR_total_ versus temperature (20–50 °C) for the sensor probe, where the RIs of the glycerin solutions with different concentration at 20 °C are 1.34, 1.35, 1.36, 1.37, 1.38, and 1.39, respectively.

Obviously, it is desirable to separate the desired TDR_sensor_ of the POF probe from the measured TDR_total_. The relationship between TDR_total_ and the temperature is shown in Figure 10 below.

**Figure 11:**
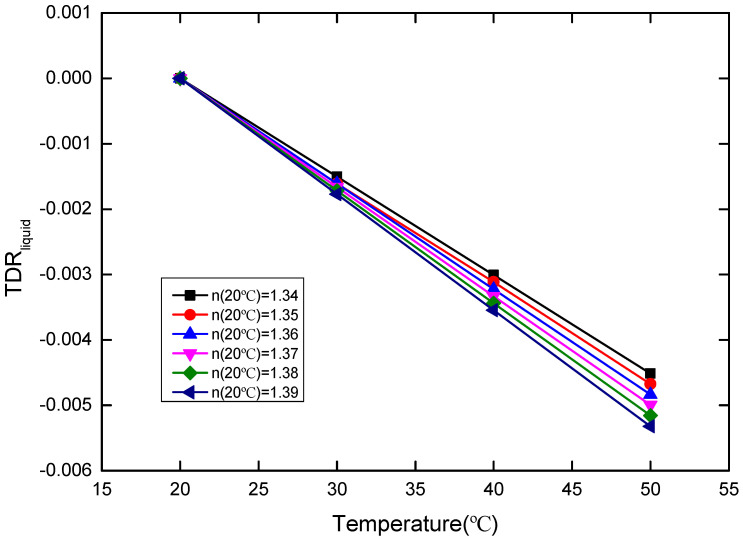
TDR_liquid_ versus temperature (20–50 °C) for the sensor probe, where the RIs of the glycerin solutions with different concentration at 20 °C are 1.34, 1.35, 1.36, 1.37, 1.38, and 1.39, respectively.

**Figure 12:**
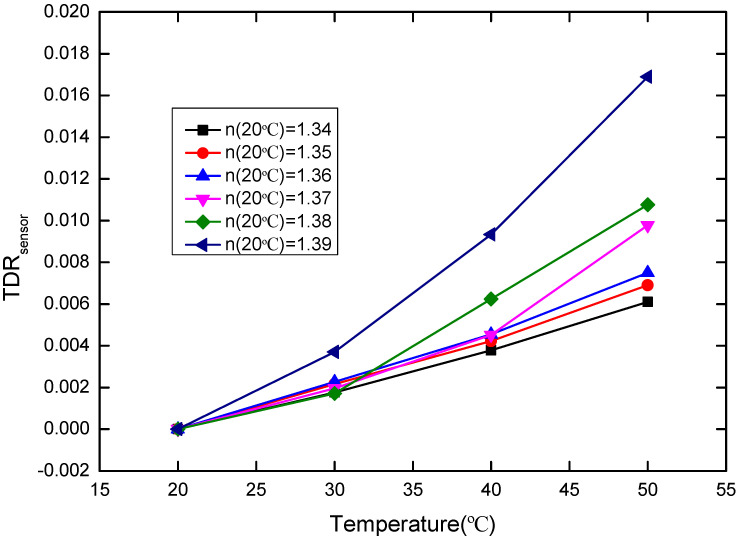
TDR_sensor_ versus temperature (20–50 °C) for the sensor probe, where the RIs of the glycerin solutions with different concentration at 20 °C are 1.34, 1.35, 1.36, 1.37, 1.38, and 1.39, respectively.

**Figure 13:**
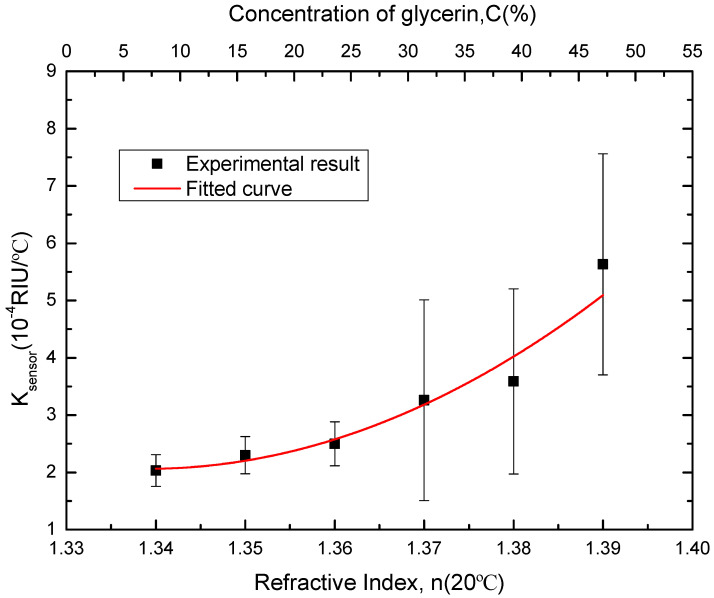
k_sensor_ versus concentration of glycerin solution and RI of measured liquid at 20 °C for the sensor probe.

To confirm the thermo-optic effect on glycerin solutions with different concentrations, the weighted arithmetic average of glycerin and water is adopted to study the RI of glycerin solution:

k_liquid_ = [Ck_1_ + (100% − C)k_2_] (7)

where k_1_ = −2.15 × 10^−4^/°C and k_2_ = −1.45×10^−4^/°C are thermo-optic coefficients of glycerin and water, respectively. C is the volume concentration of glycerol, which can be obtained by n = 1.333 + 0.13 C. Based on the Equations (4) and (7), the k_liquid_ can be obtained. Then, we have the TDR_liquid_:

TDR_liquid_ = k_liquid_ (T − 20) (8)

where T is the temperature. The relationship between TDR_liquid_ and the temperature is shown in Figure 11 below. According to Equation (6), TDR_sensor_ of the POF probe can extracted from TDR_total_, which is shown in Figure 12. It can be seen that the TDR_sensor_ of the POF probe shows the almost linear variation with the temperature increment, which facilitates the temperature correction. Here, we define the slope of TDR_sensor_ with temperature variation as:

(9) $$k_{\mathrm{sensor}}=\frac{\partial }{\partial T}\;{\mathrm{TDR}}_{\mathrm{sensor}}$$

where T is the temperature. Accordingly, we could have $k_{\mathrm{sensor}}$ as a function of the refractive index, which is shown in Figure 13. The RI deviation of the sensor at temperature T allows for correction should be:

*n*(T) = *n*(20 °C)−*k*(C)_sensor_∙(T − 20 °C) (10)

where *n*(T) and *n*(20 °C) are the actual RIs of the measured liquid at temperatures T °C and 20 °C, respectively, and *n*(20 °C) = 1.33 + 0.13C. With above analysis, the RI correction for fabricated sensor at different temperatures can be obtained. Of note, the unideal *k*_sensor_ at higher RI ranges may bring error to the temperature correction. It could be improved by optimizing the measurement setup or adopting reference sensing.

To clearly demonstrate the property of the proposed POF sensor, its RI sensing performance is compared to that of other reported works, as shown in Table 3 below. Within a RI detection range from 1.33 to 1.39, this double side-polished sensor shows the largest sensitivity compared to other sensor structures. The RI that can be detected is not as high as reported works. The relatively narrower linear detection range mainly results from the double side-polished structure. Though the interaction between light and analyte is enhanced, the saturation is more likely to happen, which limits the output response to the RI change of analyte.

## 5. Conclusions

In this work, a U-shaped double side-polished POF is proposed and experimentally demonstrated. The thermal setting method is used to fabricate the U-shaped and double side-polished probe. The symmetrical polishing on both sides of the POF offers better interaction between the liquid analyte and POF core. When the bending radius, polished depth, and deviation angle are optimized to 3 mm, 400 µm, and 60°, respectively, a sensitivity of 1541%/RIU within the RI range from 1.33–1.39 can be obtained. Stable RI sensing is able to be implemented from 20 to 50 °C. The proposed POF sensor promises good potential in liquid RI detection.

## Figures and Tables

**Table 1:** Sensitivity and resolution results from Figure 6.

Probe Polished Angle	σ_a_	Sensitivity (%/RIU) (1.33–1.39)	Resolution (RIU)	Correlation Coefficient (R^2^)
D = 0	4.31 × 10^−3^	417	3.1 × 10^−3^	0.9818
α = 30°	2.72 × 10^−3^	783	1.04 × 10^−3^	0.9927
α = 45°	5.28 × 10^−3^	1167	1.36 × 10^−3^	0.9827
α = 60°	3.72 × 10^−3^	1417	7.89 × 10^−4^	0.9778
α = 70°	4.37 × 10^−3^	1250	1.05 × 10^−3^	0.9578
α = 90°	5.18 × 10^−3^	1000	1.55 × 10^−3^	0.9629

**Table 2:** Sensitivity and resolution results from Figure 7.

Polished Depth (μm)	σ_a_	Sensitivity (%/RIU) (1.33–1.39)	Resolution (RIU)	Correlation Coefficient (R^2^)
D = 100	2.59 × 10^−3^	808	9.61 × 10^−4^	0.973
D = 200	4.17 × 10^−3^	1400	8.93 × 10^−4^	0.999
D = 300	3.72 × 10^−3^	1417	789 × 10^−4^	0.9978
D = 400	2.75 × 10^−3^	1541	5.35 × 10^−4^	0.9662
D = 500	2.66 × 10^−3^	1458	5.47 × 10^−4^	0.9919
D = 600	6.11 × 10^−3^	1245	1.47 × 10^−3^	0.9964

**Table 3:** Performance comparison of double side polished POF RI sensor with reported POF RI sensors.

Configuration	Sensitivity	Range	Resolution	Ref.
Macrobending Tapered	937%/RIU	1.33–1.41	7.11 × 10^−4^	14
Twisted Tapered	1700%/RIU3496%/RIU	1.37–1.411.41–1.44	-	15
U-shaped	864%/RIU	1.33–1.44	3.3 × 10^−4^	19
Side-Polished Macrobending	981%/RIU	1.33–1.44	-	17
This work	1541%/RIU	1.33–1.39	5.35 × 10^−4^	
